# Cesarean scar pregnancy: A case report with surgical management after initially effective conservative treatment

**DOI:** 10.1016/j.ijscr.2019.11.002

**Published:** 2019-11-06

**Authors:** Ioannis Tsakiridis, Ioannis Chatzikalogiannis, Apostolos Mamopoulos, Themistoklis Dagklis, Georgios Tsakmakidis, Apostolos Athanasiadis, Ioannis Kalogiannidis

**Affiliations:** Third Department of Obstetrics and Gynaecology, Faculty of Medicine, Aristotle University of Thessaloniki, Greece

**Keywords:** Cesarean scar pregnancy, Ectopic, Surgical, Conservative, Management

## Abstract

**Introduction:**

The incidence of cesarean scar pregnancies (CSPs) is increasing, possibly due to higher rates of cesarean sections. We report a case of a CSP with severe bleeding following initial conservative management, that was eventually treated surgically.

**Presentation of the case:**

A 33-year old woman with history of one cesarean section presented with abdominal pain and amenorrhea and was diagnosed with CSP. Conservative management with methotrexate was chosen and the treatment was regarded as successful after the expected reduction in serum hCG levels. Four days after the hospital discharge, the patient was readmitted with hemodynamic instability and an emergency laparotomy was performed to remove the remaining trophoblastic tissue.

**Discussion:**

A CSP may present as a threatened miscarriage, or remain asymptomatic through the first trimester. Medical treatment may be successful, however a close follow-up is needed even after a reduction in serum hCG levels.

**Conclusion:**

We report a case of CSP that, following an initial apparently “successful” treatment with methotrexate, required surgical management due to heavy bleeding.

## Highlights

• Cesarean scar pregnancies represent a severe obstetric entity, which is probably associated with the increasing rates of cesarean sections.
• There is no consensus on the preferred mode of treatment for cesarean scar pregnancies.
• Even after an initially effective conservative management, surgical management may be needed for the final treatment of cesarean scar pregnancies.

## 1 Introduction

Cesarean scar pregnancies (CSPs) have a prevalence of 1/2000 pregnancies and account for 6% of ectopic pregnancies [1]. Their increasing prevalence is considered a consequence of the increasing rates of cesarean deliveries [2]. They are associated with high morbidity and mortality [3]; therefore, accurate diagnosis and effective management are of major importance. However, there is no consensus on the preferred mode of treatment or follow up, while various treatment modalities have been used so far, with different reported success rates [4]. Hence, we report a case of a CSP with severe bleeding after an initial conservative management. This case report has been written in accordance to the SCARE criteria [5].

## 2 Presentation of the case

A 33-year-old woman presented at the emergency department of Hippokration Hospital of Thessaloniki, with pelvic pain and 5-weeks’ amenorrhea. She reported no history of pelvic inflammatory disease or ectopic pregnancy, did not smoke and had never used any contraceptive method apart from coitus interruptus. She also reported a history of previous emergency cesarean delivery due to threatened fetal asphyxia during labor three years ago.

**Fig. 1:**
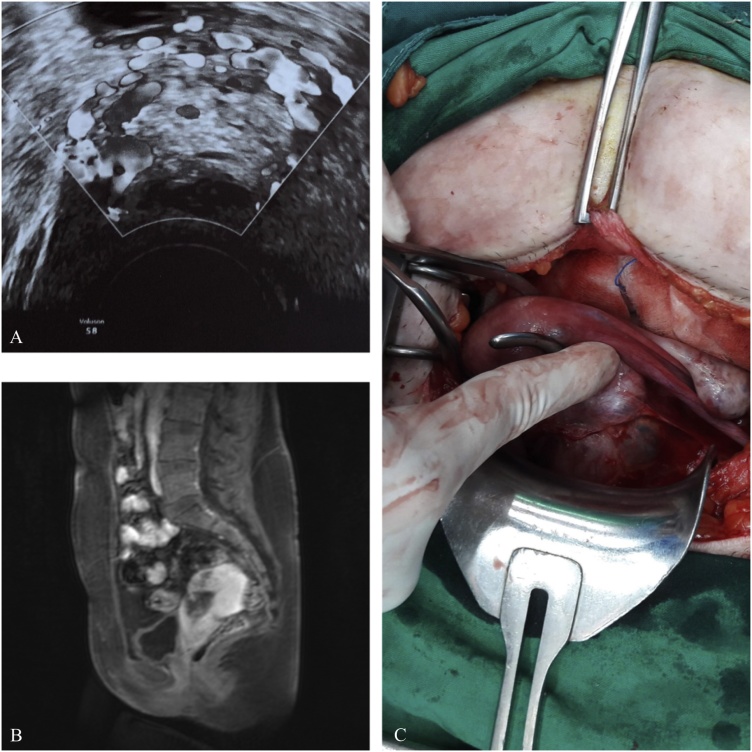
Cesarean scar pregnancy. A. Transvaginal ultrasound imaging of the cesarean scar pregnancy. B. Magnetic resonance imaging of the cesarean scar pregnancy. C. Intraoperative view of the cesarean scar pregnancy.

On physical examination, the abdomen was mildly tender without rebound tenderness, there was no vaginal bleeding, but the woman reported spotting 10 days ago. All vital signs were normal; the temperature was 36.1 °C, the heart rate was 76 beats/min, the blood pressure was 110/65 mmHg, the respiration rate was 16 breaths/min and O_2_ saturation was 100%. Serum human chorionic gonadotropin (hCG) was 2630 IU/L and hemoglobin was 13.4 g/dL. Transvaginal sonography (4–9 MHz transducer-Voluson S8, GE Healthcare, Austria) identified a gestational sac at the lower uterine segment at the level of the cesarean scar and possible trophoblastic circulation on Doppler examination (Fig. 1A). Both the endocervical canal and the endometrial cavity were empty and the endometrium measured 9 mm. Both adnexa were normal and 40 mL of free fluid was seen in the Douglas’ pouch. The suspected CSP was also confirmed by magnetic resonance imaging (MRI) (Fig. 1B).

Since all vital signs were stable and serum hCG was <5000 IU/L, medical management using systemic multidose methotrexate (MTX) was chosen and 1 mg/kg body weight of MTX was administered intramuscularly on days 1, 3, 5, and 7 combined with folinic acid (0.1 mg/kg) orally on days 2, 4, 6, and 8, as recommended [6].

During hospitalization, the patient’s vital signs remained stable and the levels of hCG dropped by more than 15% between days four (2041 IU/L) and seven (795 IU/L). Thus, medical management was considered successful and the patient was discharged on day eight and was counselled on a follow-up with weekly hCG measurements until its levels fall <5 IU/L.

Four days following the discharge (day 12), the patient presented with pelvic pain, heavy vaginal bleeding and hemodynamic instability: a heart rate of 110 beats/min, a blood pressure of 85/60 mmHg and a hemoglobin level of 6.4 g/dL. Since uterine rupture was suspected and the patient showed hemodynamic instability, an emergency laparotomy was performed and a purple mass was seen protruding through the lower uterine segment above the internal os (Fig. 1C). This was removed and the scar repaired. The patient received intraoperatively 4 units of red blood cells. During the next 2 days, all the vital signs were stable and hemoglobin levels reached 8.7 g/dL. The patient was discharged three days later. The pathology assessment of the specimen confirmed the presence of trophoblastic tissue, implanted in an area of markedly attenuated myometrium. During follow-up, serum hCG fell to non-pregnant state levels, seven days after the hospital discharge (day 22) (Table 1). The patient was advised to avoid a subsequent pregnancy for at least six months, because of MTX treatment and the repair of the uterine scar.

**Table 1:** Timeline of the total management of the cesarean scar pregnancy.

	Day 1	Day 4	Day 7	Day 12	Day 22
*Hemoglobin*	13.4 g/dL			6.4 g/dL	
*hCG*	2630 IU/L	2041 IU/L	795 IU/L		<5 IU/L
*Vital signs*	Normal	Normal	Normal	Hemodynamic instability	Normal

## 3 Discussion

The first case with a CSP was reported as early as in 1978, when a misdiagnosis of incomplete abortion led to severe hemorrhage [7]. Recently, CSPs have been classified into two types: the first type is growing inwards, with the potential of evolving to a viable fetus but with a very high risk of abnormally invasive placenta and severe bleeding and the second type is growing towards the serosal uterine surface (as identified in our case), with a high risk of rupture early in pregnancy [8].

Clinical symptoms of a CSP include vaginal bleeding and abdominal pain, while many patients are asymptomatic. Early and accurate diagnosis of this entity is of major importance as undiagnosed cases may lead to severe hemorrhage, uterine rupture and even to hysterectomy [3]. The differential diagnosis of a CSP includes threatened miscarriage, incomplete miscarriage, cervical pregnancy and malignant trophoblastic tumor [2].

Regarding the diagnosis, the findings are similar to those in other types of ectopic pregnancies: suboptimal rise of hCG, hemodynamic instability and dropping hemoglobin level due to hemorrhage [9]. The gold standard imaging method for the diagnosis of CSP is transvaginal ultrasound (86.4% sensitivity) [9] revealing an empty uterus and endocervical canal, a gestational sac at the site of the cesarean scar, a thin or absent myometrial layer and increased blood flow between the sac and the bladder (trophoblastic circulation) [10]. In addition, MRI is equally accurate with ultrasound regarding the diagnosis of CSP, but is more informative in the evaluation of scar implantation [11].

As for the management of CSP, treatment options include expectant management, administration of MTX, surgery or uterine artery embolization [12]. The expectant, the conservative and the surgical management of this condition have a success rate of up to 41.5%, 75.2% and 97.1% respectively [12]. Expectant management is an acceptable option in non-viable CSP’s and it may also be discussed in cases that appear to grow inwards, however following extensive counselling regarding the associated risks [4]. A recent systematic review of 56 cases of CSP found that live births were achieved in 73% of the cases and 25% of them were preterm [13]. In addition, more than half of cases with no fetal cardiac activity resolved during expectant management, but high (70%) rates of hysterectomy were reported overall [13]. Another meta-analysis concluded that, in cases of CSPs with fetal heart activity, expectant management is associated with a high risk of maternal morbidity, whereas it may be a reasonable option in cases with no cardiac activity [3].

Medical treatment with MTX may be performed by local injection into the sac under ultrasound guidance or by intramuscular injection, however, the trophoblast may persist in situ and cause hemorrhage [4]. A recent randomized trial compared the effectiveness of local and systemic MTX in cases of CSP and found comparable success rates [14]. In a systematic review, systemic MTX was effective only in cases with hCG levels less than 12,000 and no fetal cardiac activity [12]. Moreover, a study on local administration of MTX for the treatment of CSP reported a success rate of more than 60%, however almost one out of five patients eventually required surgical management [15]. There are also reports in the literature of cases successfully treated with a combination of local MTX and uterine artery embolization [16].

Surgical management consists of evacuation of the pregnancy by dilatation and curettage, hysteroscopic resection, or excision of the pregnancy via laparotomy, laparoscopy or transvaginally [12]. Dilatation and curettage as a treatment of CSP is highly correlated with severe complications [12]. A hysteroscopic approach is rarely used alone due to the increased need for further interventions [17]. Hysteroscopy alone may be used effectively in cases of CSPs growing inwards [18], whereas laparoscopy may be used in cases of CSPs that are growing towards the serosal uterine surface [19]. However, hemodynamic instability is a contraindication for laparoscopy and in such cases, as was in our case, a laparotomy should be performed. A resection of the CSP through a transvaginal approach has also been reported as a successful treatment modality, however more research is needed in order to establish this novel method [12].

## 4 Conclusion

This was a case of CSP that, following an initial apparently “successful” treatment with MTX, eventually required surgical management due to heavy bleeding from the persisting trophoblastic tissue. Our case highlights the need for close follow-up of medically treated cases in order to minimize the risk of rupture and preserve fertility. Due to the worldwide “epidemic” of cesarean section rates, healthcare providers should always include CSP in the differential diagnosis of women with a history of previous cesarean section presenting in the first trimester of pregnancy with pelvic pain and vaginal bleeding.

## Funding

This research did not receive any specific grant from funding agencies in the public, commercial, or not-for-profit sectors.

## Ethical approval

This article does not contain any personal information that can lead to the identification of the patient.

## Consent

Written informed consent was obtained from the patient for publication of this case report and accompanying images. A copy of the written consent is available for review by the Editor-in-Chief of this journal on request

## Author contribution

Tsakiridis Ioannis: Paper design, data collection, paper writing.

Chatzikalogiannis Ioannis: Paper design, data collection, paper writing.

Mamopoulos Apostolos: Paper review, Picture preparation.

Dagklis Themistoklis: Picture preparation, paper review.

Tsakmakidis Georgios: Paper design, paper review.

Athanasiadis Apostolos: Paper review.

Kalogiannidis Ioannis: Paper design, data collection, paper review.

## Registration of research studies

N/A.

## Guarantor

Ioannis Tsakiridis, Ioannis Kalogiannidis.

## Provenance and peer review

Not commissioned, externally peer-reviewed.

## Declaration of Competing Interest

The authors have no conflict of interest to declare.
